# New SigD-regulated genes identified in the rhizobacterium *Bacillus amyloliquefaciens* FZB42

**DOI:** 10.1242/bio.021501

**Published:** 2016-10-26

**Authors:** Ben Fan, Yu-Long Li, Aruljothi Mariappan, Anke Becker, Xiao-Qin Wu, Rainer Borriss

**Affiliations:** 1 Co-Innovation Center for Sustainable Forestry in Southern China, College of Forestry, Nanjing Forestry University, Nanjing 210037, China; 2 Institut für Biologie/Bakteriengenetik, Humboldt Universität zuBerlin, Chausseestrasse 117, Berlin D-10115, Germany; 3 LOEWE Center for Synthetic Microbiology, Marburg an der Lahn, Philipps-Universität Marburg, Marburg 35037, Germany; 4 Fachgebiet Phytomedizin, Albrecht Daniel Thaer Institut für Agrar- und Gartenbauwissenschaften, Lebenswissenschaftliche Fakultät, Humboldt Universität zu Berlin, Berlin 14195, Germany

**Keywords:** SigD, Sigma factor, *Bacillus amyloliquefaciens*, FZB42, Microarray, Non-isotopic northern blot, Soil extract

## Abstract

The alternative sigma factor D is known to be involved in at least three biological processes in Bacilli: flagellin synthesis, methyl-accepting chemotaxis and autolysin synthesis. Although many *Bacillus* genes have been identified as SigD regulon, the list may be not be complete. With microarray-based systemic screening, we found a set of genes downregulated in the *sigD* knockout mutant of the plant growth-promoting rhizobacterium *B. amyloliquefaciens* subsp. *plantarum* FZB42. Eight genes (*appA*, *blsA*, *dhaS*, *spoVG*, *yqgA*, *RBAM_004640*, *RBAM_018080* and *ytk*) were further confirmed by quantitative PCR and/or northern blot to be controlled by SigD at the transcriptional level. These genes are hitherto not reported to be controlled by SigD. Among them, four genes are of unknown function and two genes (*RBAM_004640* and *RBAM_018080*), absent in the model strain *B. subtilis* 168, are unique to *B. amyloliquefaciens* stains. The eight genes are involved in sporulation, biofilm formation, metabolite transport and several other functions. These findings extend our knowledge of the regulatory network governed by SigD in *Bacillus* and will further help to decipher the roles of the genes.

## Abstract (precis)

**Summary:** New SigD-regulated genes were identified in the plant growth-promoting rhizobacterium *Bacillus amyloliquefaciens* FZB42, which is closely related to the model organism *B. subtilis*.

## INTRODUCTION

In prokaryotes, which lack a nuclear membrane, transcription and translation take place simultaneously. Contrary to the multilevel regulations happening almost equivalently in eukaryotic cells, the control of gene expression in bacteria occurs primarily at the level of transcription. Usually, the holoenzyme of a bacterial RNA polymerase (RNAP) consists of two parts: the catalysing core enzyme and an additional σ factor, which permits the holoenzyme to anchor on certain promoter sites and initiate transcription from the regions. That is, the σ subunit associates with the core RNAP and determines most, if not all, of the specificity of bacterial RNAPs to their cognate promoter.

Much of the biochemistry of bacterial RNAPs was derived from *Escherichia coli*, which is assumed to be directly applicable to the *B. subtilis* enzymes. However, the RNAPs from the two species are not identical. For example, *B. subtilis* has at least 17 different σ factors (Yoshimura et al., 2004), seven of which (σ^M^, σ^V^, σ^W^, σ^X^, σ^Y^, and σ^Z^) belong to the members of the extracytoplasmic (ECF) subfamily. The plant root-associated *B. amyloliquefaciens* FZB42 is a phylogenetically close relative of *B. subtilis* (Borriss et al., 2011). FZB42 has strong ability to promote plant growth and suppress plant pathogens, and thus has been extensively studied as a paradigm of rhizobacteria (Koumoutsi et al., 2007; Schneider et al., 2007; Chen et al., 2007, 2009a,b; Chen, 2010; Borriss et al., 2011; Fan, 2011; Fan et al., 2012a,b, 2015; Liu et al., 2013; Scholz et al., 2014; Wu et al., 2015). FZB42 encodes 16 σ factors, six of which (σ^M^, σ^V^, σ^W^, σ^X^, YlaC, and a unique putative σ factor RBAM_006770) are predicted to be ECF sigma factors (Chen et al., 2007).

The alternative sigma factor D (σ^D^) of *B. subtilis* was identified in 1988 (Helmann et al., 1988). σ^D^ is around 28 kDa and peaks in expression at late exponential phase (Helmann et al., 1988). The *sigD* gene of *B. subtilis* locates at the end of the *fla-che* operon comprising over 30 genes. Based on known information, σ^D^ plays a fundamental role in the transcription of the genes for flagellin (Mirel and Chamberlin, 1989), methyl-accepting chemotaxis receptor proteins (Marquez et al., 1990), and autolysin synthesis (Marquez et al., 1990; Kuroda and Sekiguchi, 1993). The unique binding motif of σ^D^ [TAAA(-35)-N15-GCCGATAT(-10)] has been summarised, which allows researchers to detect those genes with an upstream σ^D^-recognised sequence (Helmann, 1991). For instance, two genes (*degR* and *epr*) with such a σ^D^*-*like promoter were thereby identified (Helmann, 1991). In addition, some *B. subtilis* genes were identified to be SigD-dependent by DNA microarray and northern blot (Serizawa et al., 2004); these genes were also preceded by a σ^D^-recognized promoter.

More than 50 monocistronic and polycistronic transcription units are known as being controlled by SigD in *B. subtilis* (Mader et al., 2012). However, the list of σ^D^-regulated genes may be incomplete, especially in other *Bacilli* apart from *B. subtilis*. In this work we used a two-coloured microarray system, quantitative PCR (qPCR), and northern blot, to identify potential members of the SigD regulon in *B. amyloliquefaciens* FZB42. Our results obtained contribute to the understanding of regulatory mechanisms of *B. amyloliquefaciens* subsp*. plantarum*, a group of soil bacteria with immense ecological importance, and their interaction with plants.

## RESULTS

### Candidate SigD-dependent genes derived from microarray experiments

**Fig. 1.**
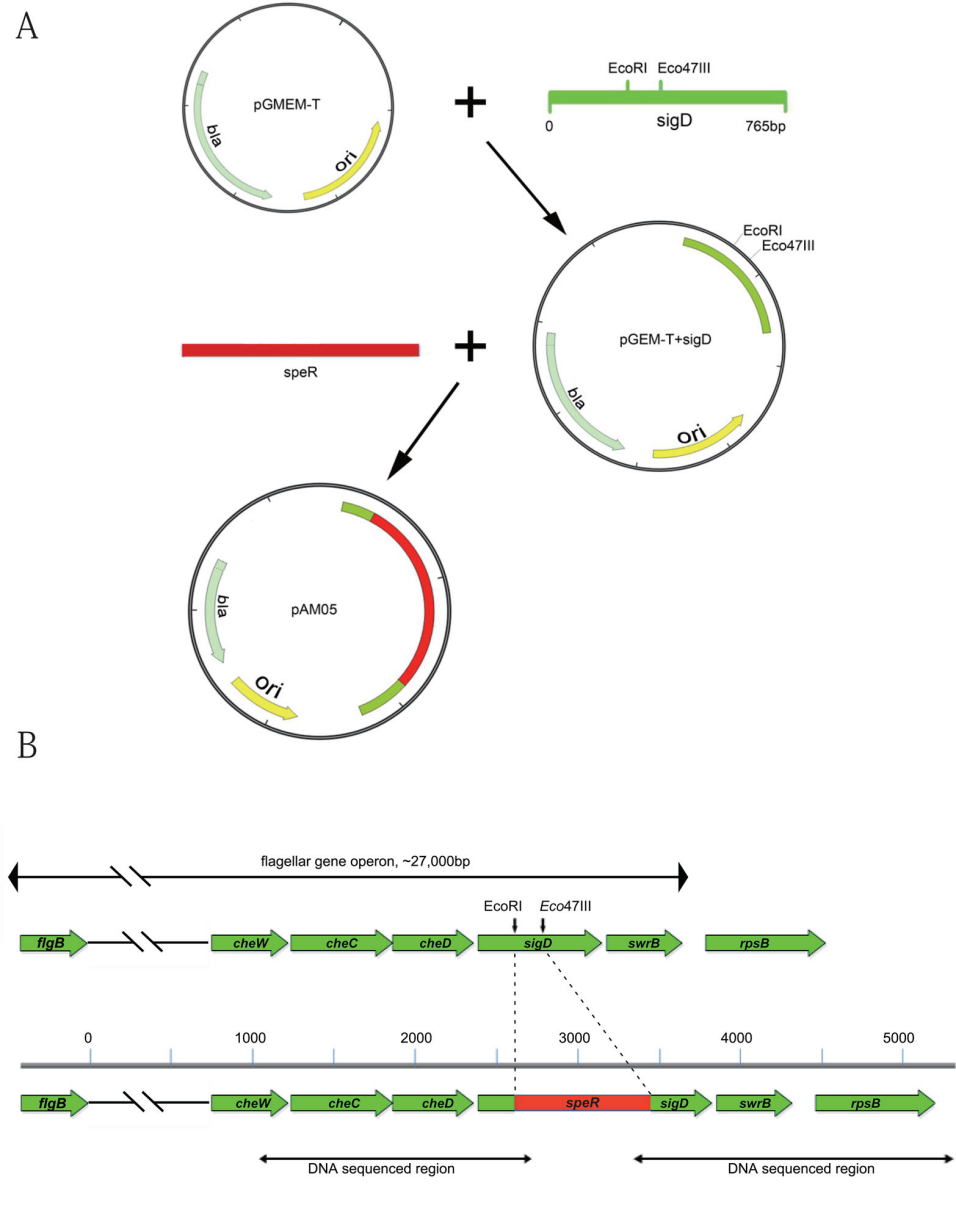
**Construction for *sigD* deletion mutant of *B. amyloliquefaciens* FZB42.** (A) The integrating plasmid was constructed as follows: the *sigD* gene fragment was amplified from the genome DNA of FZB42 and inserted into vector pGEM-T, yielding a new plasmid pGEM-T_sigD. The spectinomycin-resistance gene was amplified and cloned between the *Eco*47III and *Eco*RI restriction sites of pGEM-T_sigD, thus obtaining pAM05 carrying *sigD*::*speR*. (B) After transformation, the *sigD* gene was disrupted as indicated. The *sigD* is located at the penultimate locus of the flagellar gene operon which contains 32 genes. The gene local context of *sigD* is shown in scale. The two lines at the bottom cover the regions where DNA sequencing was performed to confirm the intactness of the flanking genes of *sigD* in the mutant.

The *sigD* gene of *B. amyloliquefaciens* FZB42 was successfully deleted using the strategy shown in Fig. 1A. The deletion mutant was firstly confirmed by colony PCR and the feature of filamentous cell chains that are non-motile when grown in LB. DNA sequencing showed that the sigD gene was precisely disrupted while their flanking regions kept intact (Fig. 1B). Total RNAs were extracted from three cultures of FZB42 wild type and the *ΔsigD* mutant, respectively. Another experiment was independently performed from which additional three pairs of cultures were similarly collected for RNA extraction. The six pairs of transcriptomes were compared using the two-colour microarray system as previously described (Fan et al., 2012a,b). The raw data of microarray experiments were deposited in the ArrayExpress database under the accession numbers: E-MTAB-4876. A False Discovery Rate (FDR) significance test was performed for the data.

Using an adjusted *P*-value of <0.01 and an arbitrary filter fold change >3.0, that is, the transcriptional level of a gene in the wild type was three times more than in the *sigD* deletion mutant, we identified a total of 41 genes as candidates of genes directly or indirectly regulated by SigD (Table 1). Except *hag*, the gene involved in motility and chemotaxis, all other identified genes have not been previously described as members of the SigD regulon. Nearly 60% (24 genes) of the 41 genes encode a hypothetical protein. Additionally, five (*pznF*, *pznH*, *dfnF*, *dfnJ* and *dfnL*) of the 41 genes are involved in production of two antimicrobial compounds (Table 1): the first two genes are involved in plantazolicin synthesis (Scholz et al., 2011); the latter three genes belong to a gene cluster encoding modular polyketide synthase for difficidin. A strong ability to produce multiple antimicrobial metabolites is a remarkable feature of FZB42 (Chen et al., 2007, 2009a,b), for which it has been developed as biocontrol agents to benefit plant growth.

**Table 1.**
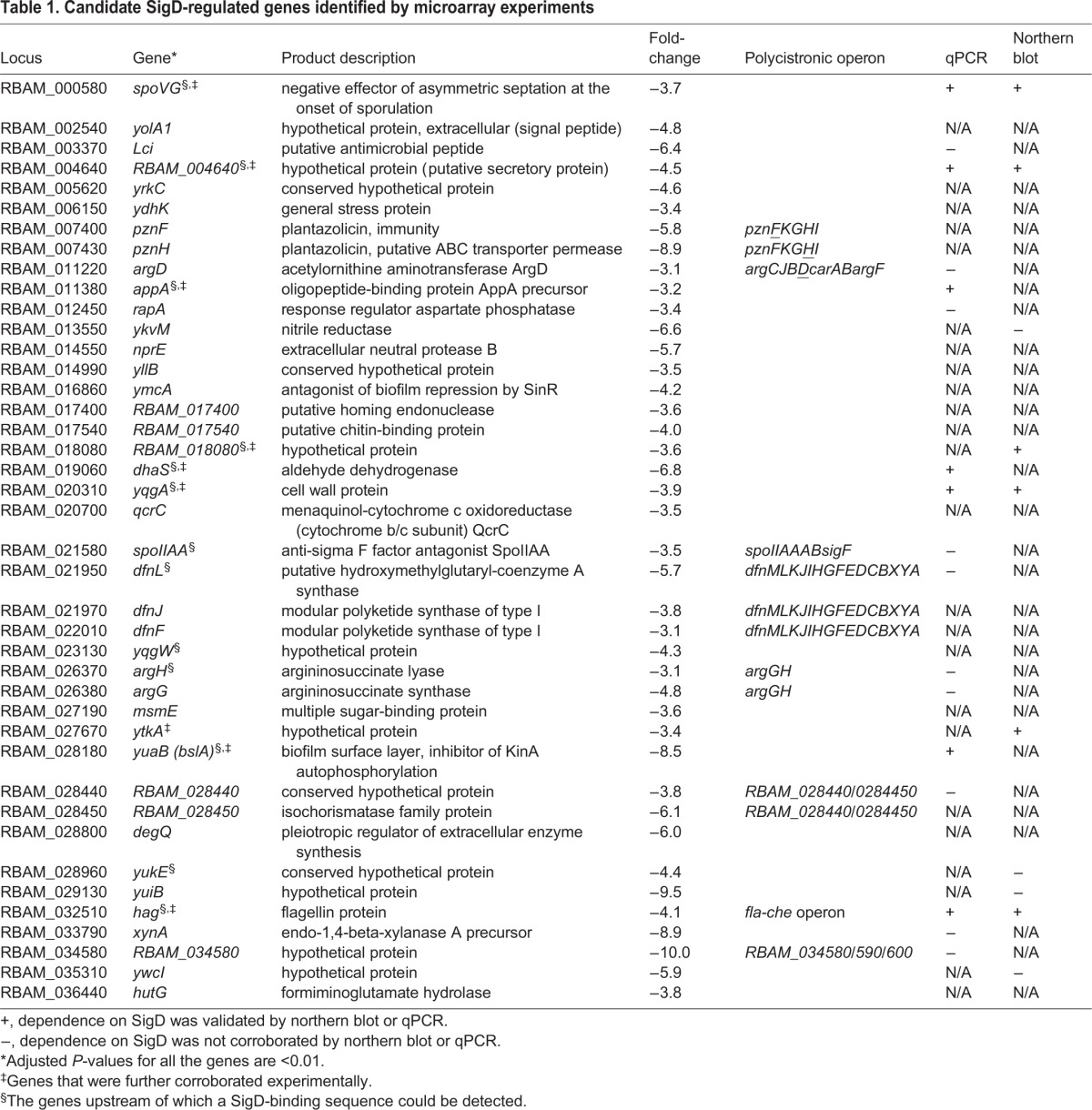
**Candidate SigD-regulated genes identified by microarray experiments**

Ten genes (*pznF*, *pznH*, *argD*, *difL*, *difJ*, *difF*, *argH*, *argG*, *RBAM_028440*, *RBAM_028450*) of the 41 candidates were assigned to five multicistronic operons (Table 1), which were either known previously or predicted by DOOR (the Database of prOkaryotic OpeRons) (Mao et al., 2009). Since an operon is transcribed into one transcript, we selected only one gene in each operon for further analysis. Furthermore, we examined the ∼400-bp promoter region upstream of the transcription start site (TSS) of each candidate gene (Fan et al., 2015) for potential sigma factor recognizing sequences (Sierro et al., 2008). Taken together, we chose 23 candidate genes (Table 1) for subsequent experimental validation of their dependence on SigD.

### Validation of SigD-regulated genes by quantitative PCR

Quantitative PCR (qPCR) is a convenient approach to compare transcriptional levels of a gene across samples, especially useful for the detection of very long transcripts, e.g. those generated from a polycistronic operon. Therefore, we firstly used qPCR to examine 17 candidate SigD-regulated genes. RNAs were extracted from LB cultures of the FZB42 wild type and the *ΔsigD* mutant collected at 4 h post-inoculation (hpi) (Fig. S1), corresponding to the end of the exponential phase when SigD is known to accumulate abundantly. The microarray experiments were originally designed to support our study on plant-microbe interaction (Fan, 2011; Fan et al., 2012a,b). Since the soil extract used in 1C medium for microarray experiments was no longer available, we decided to turn to LB in our validation experiments. Theoretically, a gene should be more robustly controlled by SigD if this could be confirmed in two different media.

**Fig. 2.**
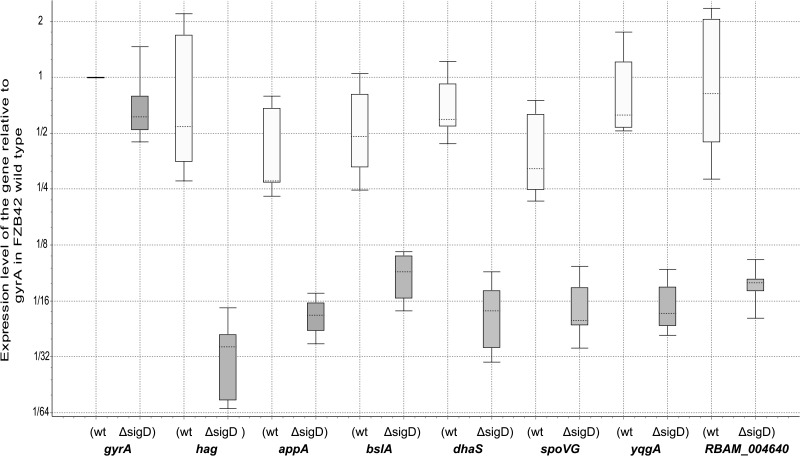
**Relative gene expression in the *B. amyloliquefaciens* FZB42 wild type and it *sigD* deletion mutant revealed by qPCR.** The expression of the gene in the *B. amyloliquefaciens* FZB42 wild type and in the *sigD* deletion mutant was quantified by real-time PCR and visualized by the software REST 2009 (Pfaffl, 2001; Pfaffl et al., 2002). The house keeping *gyrA* was used as inner control to normalize the data. All expression levels were calculated relative to that of *gyrA* in the wild type. The boxes represent the distance between the 25th and the 75th percentile. The lines in the boxes represent the median gene expression. Whiskers represent the minimum and maximum observations. Three biological replicates and three technical replicates for each biological replicate were used (*n*=9).

Three biological replicates were performed for the comparison. The *hag* gene was amplified as positive control and *gyrA* was used as inner control and negative control. As expected, while *hag* showed significant expression difference between the wild type and the *Δ*sigD mutant (Fig. 2), no significant difference could be detected in *gyrA* expression between the two strains. In total, six genes (*appA*, *blsA*, *dhaS*, *spoVG*, *yqgA* and *RBAM_004640*) were validated by qPCR to be SigD-regulated (Fig. 2). Three of them (*spoVG*, *yqgA* and *RBAM_004640*) were also confirmed by northern blot and thus described in later paragraphs.

**Fig. 3.**
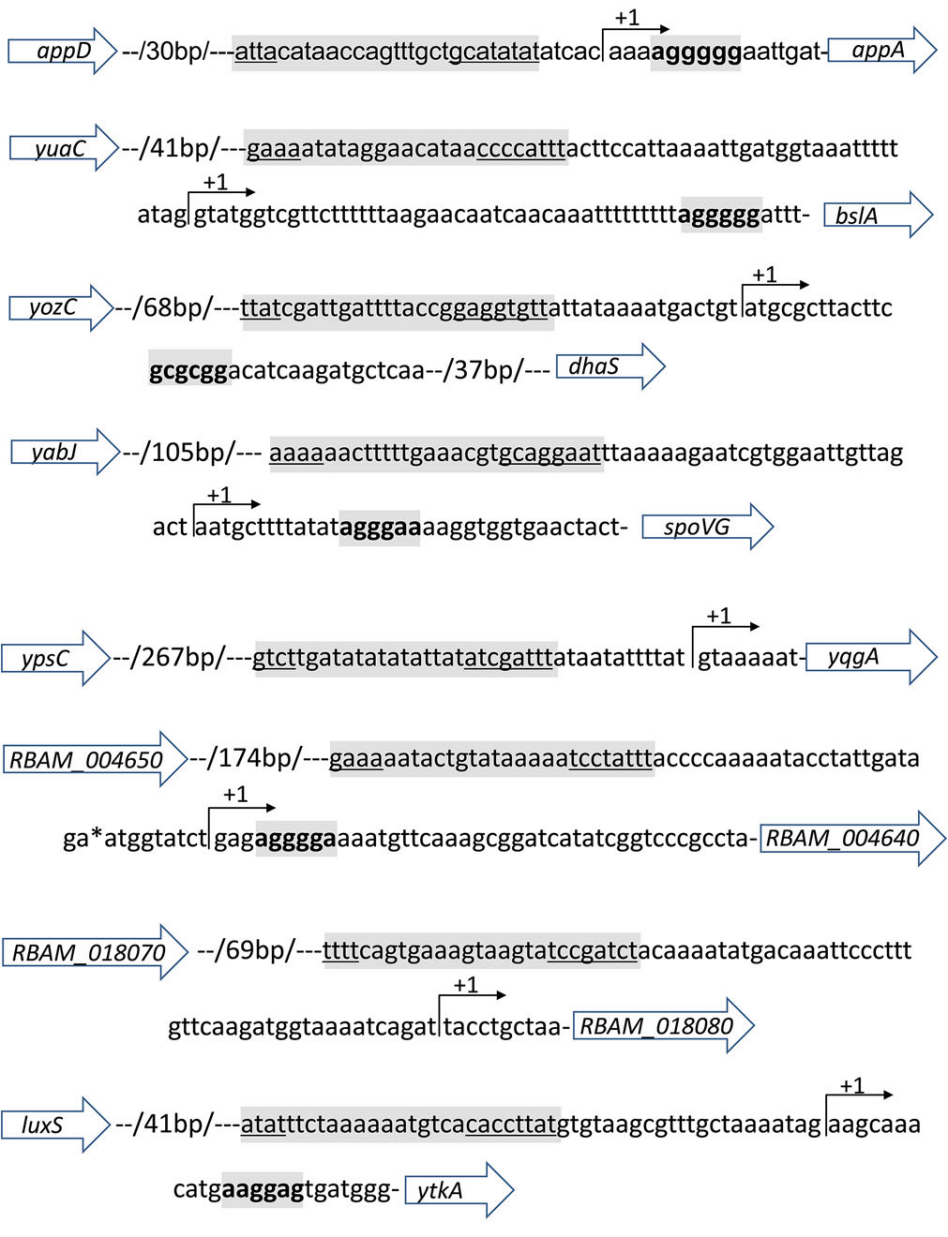
**TSSs and putative SigD-binding sites of the SigD-dependent genes identified.** All of the TSSs and SigD-binding sites of the SigD-dependent genes identified in this study are summarized. The TSS information is obtained from Fan et al. (2015). The location of a TSS is indicated by a bent arrow. The putative SigD-binding sites are indicated in grey and the −35 region and the −10 region were underlined. The ribosome binding sites are indicated in bold letter and in grey.

The first gene confirmed by qPCR to be controlled by SigD is *appA* (Fig. 2). The gene *appA* is a member of the operon *appD-appF-appA-appB-appC*, responsible for an oligopeptide ABC transporter in *B. subtilis*, and *appA* is assumed to encode the peptide-binding protein (Koide and Hoch, 1994). The operon is controlled by CodY, ScoC and TnrA. A putative *sigA* promoter was designated upstream of *appD*, the first gene of this operon (Koide and Hoch, 1994). However, we detected an internal TSS (Fig. 3) upstream of *appA* within the operon from our previous study (Fan et al., 2015). A SigD-binding motif was detected upstream of this TSS (Fig. 3). Taken together, these results suggest that transcription of *appA* is governed by SigD and starts from an inner TSS within the *app* operon (Sharma et al., 2010).

Secondly, we validated whether *bslA* (*yuaB*) is regulated by SigD (Fig. 2). BslA is an amphiphilic protein involved in forming a hydrophobic surface layer of biofilms. The expression of *bslA* is regulated by multiple regulators such as DegU, AbrB and LutR (Verhamme et al., 2009; Ostrowski et al., 2011; Irigul-Sonmez et al., 2014), but it has not been reported which sigma factor controls the transcription of *bslA*. We also found a SigD-binding motif upstream of the TSS of *bslA* (Fig. 3), suggesting that *bslA* is directly controlled by SigD.

The third gene we confirmed with qPCR is *dhaS* (Fig. 2). As a homolog of aldehyde dehydrogenases, DhaS is able to catalyse 3-hydroxypropionic acid (3-HPA) synthesis from 3-hydroxypropionaldehyde (Su et al., 2015). DhaS is similar to indole-3-acetaldehyde dehydrogenase from *Ustilago maydalis* (Basse et al., 1996), and is thought to be involved in the biosynthesis of plant growth hormone indole-3-acetic acid (IAA) in *B. amyloliquefaciens* (Idris et al., 2007; Shao et al., 2015). The presence of a SigD binding motif upstream of the TSS of *dhaS* (Fig. 3) suggests that *dhaS* is directly transcribed by SigD.

### Validation of SigD-regulated genes by northern blot

**Fig. 4.**
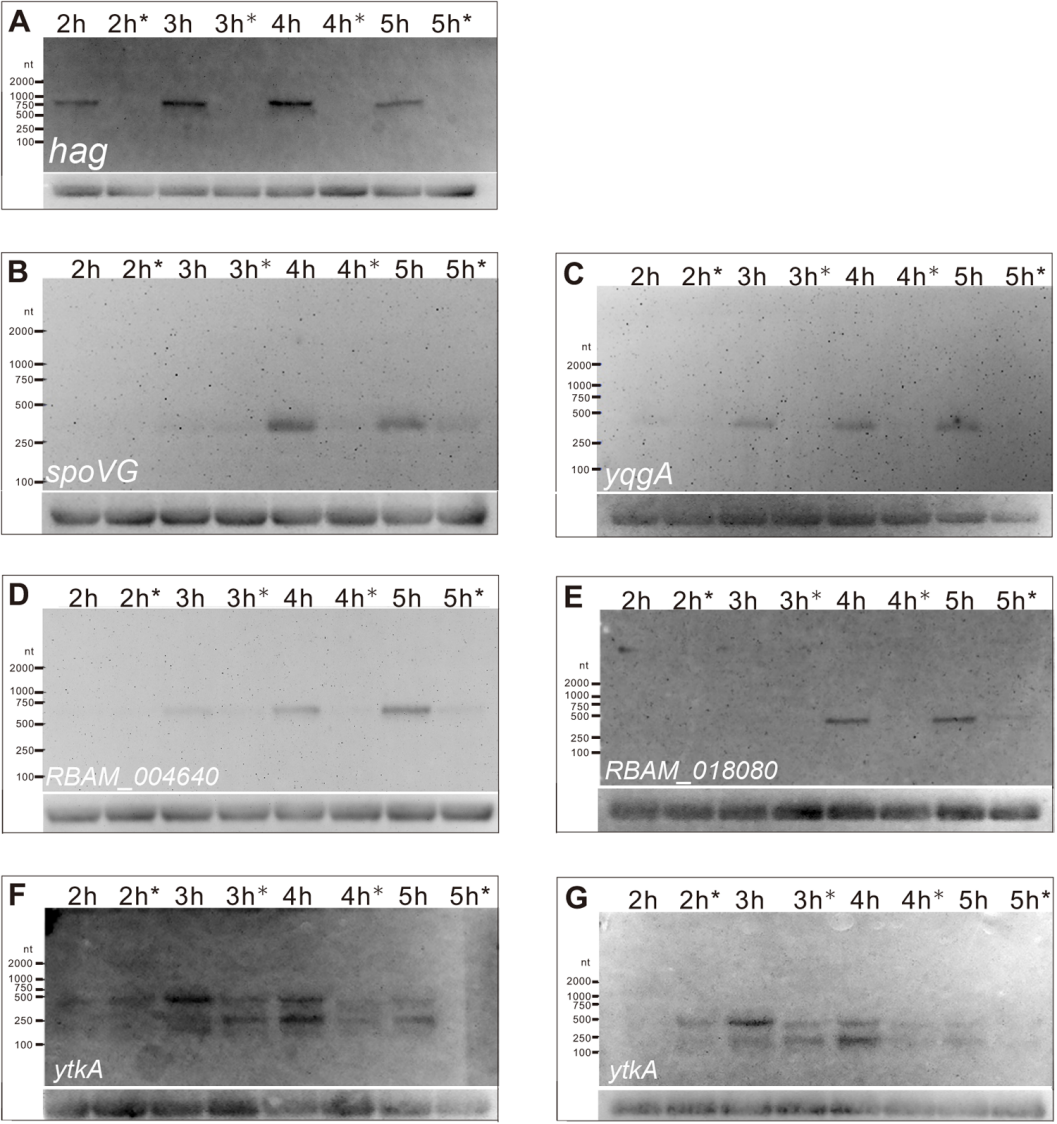
**Expression of the sigD-regulated genes.** (A) *hag*, (B) *spoVG*, (C) *yqgA*, (D) *RBAM_004640*, (E) *RBAM_018080* and (F,G) *ytkA* in *B. amyloliquefaciens* FZB42 wild type and its *sigD* deletion mutant, as revealed by northern blot. 2 h, 3 h, 4 h and 5 h represent time after inoculation at which cultures were sampled for RNA preparation. The hours with an asterisk are samples from the *sigD* deletion mutant; those without an asterisk are from wild type. 5S RNA was detected as loading control. nt, nucleotide.

Northern blotting provides a robust semi-quantitative method to compare gene expression across samples over time. To verify our qPCR results, we further tested the expression of three genes, which had already been validated by qPCR, by using northern blot. In addition, another four genes harbouring putative upstream SigD binding motifs were also included in this analysis. The total RNAs were extracted from the cultures sampled at four time points (2, 3, 4 and 5 hpi) (Fig. S1). We used *hag* as a positive control (Fig. 4A) and 5S rRNA the as negative and loading control. As a result, we confirmed all the three genes (*spoVG*, *yqgA* and *RBAM_004640*) previously validated by qPCR, as well as two more genes, *RBAM_018080* and *ytkA*, to be controlled by SigD.

Initially, we confirmed by northern blot that *spoVG* is controlled by SigD (Fig. 4B). Interestingly, *spoVG* is also known as a member of the sigH regulon (Segall and Losick, 1977). A typical SigD-binding motif was identified upstream of the TSS of *spoVG* (Fig. 3). The product of this gene, SpoVG, is known as a negative regulator of asymmetric septation during the onset of sporulation (Matsuno and Sonenshein, 1999). The expression of *spoVG* during logarithmic growth is repressed by the global transcriptional regulators AbrB (Zuber and Losick, 1987; Fürbaß et al., 1991) and SinR (Chu et al., 2006). The *spoVG* mRNA peaked at 4 hpi, corresponding to the late logarithmic growth.

Another gene, transcriptionally controlled by SigD, is *yqgA* (Fig. 4C)*.* YqgA is a cell wall-attached protein localized at cell division sites during the transition period between the exponential and the stationary phases (Hashimoto et al., 2014). YqgA localization is affected by mutations in the DL-endopeptidases (DLEPases), which are the autolysins involved in cell morphogenesis (Hashimoto et al., 2014). *yqgA* was transcribed abundantly during 3-5 hpi, which roughly matched the transition period from exponential to stationary phase. Again, a SigD­-binding motif was detected upstream of the TSS of *yqgA* (Fig. 3).

Two genes with unknown function, *RBAM_004640* and *RBAM_018080*, were also confirmed to be regulated by SigD. Both genes are unique in *B. amyloliquefaciens* subsp. *plantarum* and have no counterparts in *B. subtilis.* SigD binding sequences were also detected upstream of the TSS of *RBAM_004640* and *RBAM_018080* (Fig. 3). Transcription of these genes started either at 3 hpi (*RBAM_004640*) or at 4 hpi (*RBAM_018080*) and peaked at 5 hpi (Fig. 4D,E).

The last gene we validated with northern blot was *ytkA*, whose function is also unknown. Interestingly, we detected two transcripts, around ∼500 nt and ∼250 nt in size in the blot (Fig. 4F). For validation, we designed a second probe targeting the region 25 bp distant from the first probing sequence, for northern blot detection. With the new probe, we obtained almost the same banding pattern (Fig. 4G) and thus confirming the first probing result. Both transcripts were mostly expressed between 3 hpi and 4 hpi, and started to decrease at 5 hpi. The two transcripts showed in their expression an apparently reciprocal relationship; the larger transcript was accumulated at 3 hpi, whilst the highest concentration of the shorter transcript was at 4 hpi (Fig. 4F,G).

## DISCUSSION

In this study, we firstly performed a microarray-based screening of the transcriptome of *B. amyloliquefaciens* FZB42 for SigD-regulated genes. By qPCR and northern blot, we validated eight new genes controlled by SigD in *B. amyloliquefaciens*. In addition, SigD-recognized sites were detected upstream of TSSs of the genes. Two of the novel genes governed by SigD, *RBAM_004640* and *RBAM_018080***,** are unique in *B. amyloliquefaciens*; the other six occur in both *B. amyloliquefaciens* and *B. subtilis* (Table 1). Our findings added new members to the list of around 50 known *Bacillus* SigD regulons. Among the eight genes, four of them encode proteins with unknown function (*RBAM_004640*, *RBAM_018080*, *yqgA*, *ytkA*). Association of these genes with SigD will facilitate deciphering their functions and regulation machinery. For example, we infer that special investigations with a *ΔsigD* mutant may help to elucidate possible involvement of the genes in the biological processes known to be directed by SigD, such as chemotaxis, mobility or autolysis.

While DNA microarray offers a high throughput method for systematic identifying genes of interest, it is nearly inevitable that some false positive results will be generated by this approach (Ogura et al., 2001; Asai et al., 2003; Serizawa et al., 2004; Stephan et al., 2005). To filter the microarray result, we applied a threshold (q≤0.01 and fold-change >3.0), with which only one known SigD-dependent gene, *hag*, was detected. Although the SigD regulon of FZB42 would not be simply identical to that of *B. subtilis*, for example, 16 genes (*sivC*, *tlpC*, *ybdO*, *yjcP*, *yjcQ*, *yoaH*, *yscB*, *yvyC*, *yjcM*, *dgcW*, *ylzI*, *yoyG*, *yqaR*, *yqaS*, *yvaQ*, *smiA*) known to be controlled by SigD in *B. subtilist* are absent in FZB42, this is still a low recovery of known SigD targets. But the recovery rate can be improved; if we used a less stringent condition, e.g. q≤0.05 and fold-change >1.4, a total of 16 genes (*cheA*, *dltA*, *dltB*, *dltC*, *flgC*, *flhB*, *fliD*, *fliL*, *hag*, *lytB*, *lytF*, *swrA*, *swrB*, *yfmS*, *yojL*, *ytlR*), which have been previously reported to be controlled by SigD, can be detected. In this case, however, the higher recovery is obtained at the price of increasing the number of the total differentially expressed genes up to 542, which will logically contain a large volume of false targets. In general, there is a high level of noise in our microarray result. This may have several causes; for example, we checked the Spearman's correlation coefficients between all microarray replicates (Fig. S2). This revealed that two replicates are in low correlation with the other arrays, which may lead to less consistent data and thus the low overlap with the known SigD targets in *B. subtilis*. In addition, we used a fixed time point for culture sampling in the microarray experiment. To support our major project (Fan, 2011; Fan et al., 2012a,b), the cultures of all FZB42-derived strains were collected at an optical density of 3.0. As such, the time window of sampling may have been too narrow to detect some known SigD-dependent genes (Mirel et al., 2000). Nevertheless, the value of the microarray result for data mining does exist; in this work we focus on identifying new targets firstly by using a stringent condition to filter the microarray result. The above reasons can also account for the fact that a limited number (Table 1) of the genes identified by DNA microarray were validated with qPCR or northern blot. However, the expression of SigD-dependent genes was regulated by environmental factors like nutritional signals (Mirel et al., 2000; Serizawa et al., 2004). The medium used in microarray experiments contained soil extract, which comprises many components that may favour some otherwise unexpressed genes (Taylor, 1951). Given these reasons, we would not exclude the possibility that some more genes in Table 1 may be corroborated to be truly SigD-dependent if diverse bacterial growth conditions, including different media and more sampling times, are tested.

Notably, some genes comprised in a large operonic structure, like *pznF* and *pznH* in the pzn operon, are identified in the transcriptome while other genes from the same operon are not identified (Table 1). This can be explained by at least several reasons: (1) Not all the probes used in the microarrays will work equivalently efficiently or perfectly; (2) it is known that internal TSSs can be present in a known operon, as we mentioned in the example of *appA*; (3) some non-coding RNAs antisense to an operon can terminate the transcript and thus lead to uneven expression of the genes in the operon.

In this study, we defined the genes downregulated upon *sigD* disruption as candidate SigD-dependent genes. This does not necessarily mean that SigD directly transcribes the gene. For example, SigD transcribes the gene *degR*, which controls the activity of DegU (Mukai et al., 1992), a transcriptional regulator controlling a large array of genes. Thus, downregulation or even upregulation of gene expression resulting from DegU activity will be affected by *sigD* knockout. In this case, a typical binding motif recognized by SigD is a strong indicator for direct control of SigD. In addition, transcription of many genes is controlled by more than one regulator; thus, the effect of SigD regulation may be amplified or offset by the complexity of gene interaction networks, which must be taken into account when analysing such data.

Interestingly, six of the eight genes encode for proteins of less than 200 amino acids. The shortest one is SpoVG, which contains only 98 amino acids. Small proteins or peptides represent a reservoir of molecules, which have to some extent been ignored in the past but now receive increasing attention (Yang et al., 2011; Storz et al., 2014). Further investigation will perhaps reveal that more genes encoding small proteins are the targets of SigD.

## MATERIALS AND METHODS

### Growth conditions of bacterial strains

*B. amyloliquefaciens* FZB42 was deposited as strain 10A6 in the culture collection of *Bacillus* Genetic Stock Centre (BGSC). For microarray experiments, two independent experiments were performed, in each of which three bacterial cultures were collected for the FZB42 wild type and its *sigD* deletion mutant, respectively. Thus a total of six biological replicates were used for both strains. For the microarray experiments both strains were grown in 1C medium (3.5% pancreatic digest of casein, 1.5% papain digest of soya flour, 2.5% NaCl) supplemented with soil extract (Fan et al., 2012a,b). For northern blot and qPCR, the two strains were cultured in standard lysogeny broth (10 g peptone, 5 g yeast extract, 5 g NaCl, 1000 ml H_2_O).

### Strain construction

Deletion of the *sigD* gene, yielding mutant AM05 (*ΔsigD*::*specR*), was obtained after transformation of *Bacillus amyloliquefaciens* FZB42 with a linearized, integrative plasmid containing a spectinomycin-resistance cassette flanked by DNA regions homologous to the FZB42 chromosome (Fig. 1). The *sigD* gene fragment amplified with primers sigD_fw and sigD_rw (Table S1) was inserted into pGEM-T to create pGEM-T_sigD. The *specR* cassette was amplified with primers Spec_fw and Spec_rw (Table S1) using plasmid pIC333 as a template. The fragment was cloned between the *Eco*47III and *Eco*RI restriction sites of pGEM-T_sigD to obtain pGEM-T carrying *sigD*::*specR*. AM05 was confirmed for successful deletion by DNA sequencing and phenotypic observation and is then stored at the Strain Collection Centre of Nord Reet UG, Greiswald, Germany.

### Total RNA preparation

Total RNA preparation for microarray experiments has been described previously (Fan et al., 2012a,b). For northern blot and qPCR, the optical density of bacterial cultures was monitored throughout their growth. Cultures at 2, 3, 4 and 5 hpi (Fig. S1) were collected for total RNA preparation. The RNAs prepared from all the four time points were used for northern blot, while extra RNAs from 4 hpi were used for qPCR. Every 15 ml of the culture was quickly mixed with 3 ml stop solution (95% ethanol+5% phenol) and centrifuged at 4°C to obtain pellets. Isolation of RNA was performed using the TRIzol^®^ Max™ Bacterial RNA Isolation Kit (Thermo Fisher Scientific) according to the manufacturer's instructions.

### Microarray detection

The microarray experiments were performed as previously described (Fan et al., 2012a,b). Briefly, synthesis of first-strand cDNA, microarray hybridization and image acquisition were performed in CeBiTec, the Centre for Biotechnology at Bielefeld University. At least three RNA samples prepared independently were used as biological replicates. In all comparisons, dye-swap was carried out to minimize the effect of dye biases. The obtained transcriptomic data were analysed with EMMA 2.8.2 software (Dondrup et al., 2009). The raw data were normalized by the method of LOWESS (locally weighted scattered plot smoothing). Significance testing was performed by the method of FDR control (Benjamini and Hochberg, 1995; Roberts and El-Gewely, 2008). The microarray result of this work is also partly available in the dissertation Fan (2011).

### Northern blot

After DNase digestion, 8 μg total RNA was denatured for 5 min at 95°C in RNA loading buffer (95% formamide, 0.1% xylene cyanole, 0.1% Bromophenol Blue and 10 mM EDTA). The denatured RNA was separated on a 1.8% RNA agarose gel under denaturing conditions (1×MOPS buffer pH 7.0 containing 10% formaldehyde) before being transferred onto Amersham Hybond-N+ membranes (GE Healthcare) at 50 V for 1 h at 4°C. 5S RNA was detected as loading control. All oligonucleotides used for transcript detection are listed in Table S1. These oligos were digoxigenin-labelled at their 3′ end using DIG oligonucleotide tailing kit (Roche) according to the manufacturer's instructions. Briefly, the labelled oligos were hybridized to membranes overnight at 42°C, before washing with 2× SSC/0.1% SDS, 1×SSC/0.1%SDS and 0.5×SSC/0.1% SDS for 10 min each. Subsequently, the membrane was blocked for 1 h at room temperature in 1× blocking reagent and then incubated with α-digoxigenin conjugated with alkaline phosphatase for 30 min. After rinsing twice, each for 20 min in wash buffer, detection was performed with chemiluminescent substrate CDP-Star diluted 1:100 in detection buffer. Signals were recorded with the imager (Proteinsimple, Fluorchem Q).

### Quantitative PCR

The isolated RNAs were digested with DNase to avoid possible trace DNA contamination. After ethanol precipitation RNA pellets were resuspended in 300 μl RNase-free H_2_O. Using random hexamers the first strands of cDNA were amplified by reverse transcription using PrimeScript™ RT Master Mix (TaKaRa). Real-time PCR was performed with 7500 Fast Real-Time PCR System (ABI, USA) and SYBR^®^*Premix Ex* Taq™ kit (TaKaRa), according to the manufacturer's instructions. The house-keeping gene *gyrA* was used as an internal control. For each gene, three technical replicates were carried out for each of three biological replicates. Oligonucleotides used were listed in Table S1. Quantification was analysed based on the threshold cycle (Ct) values as described by Pfaffl (2001). The data were visualized using the software REST 2009 (Pfaffl et al., 2002).
